# Implementing evidence-based practices for urinary leakage prevention in ICU patients with indwelling catheters: A JBI-guided baseline review

**DOI:** 10.1371/journal.pone.0351406

**Published:** 2026-07-10

**Authors:** Hong Bian, Zhiyin Zhou, Jingjing Yan, Zhengyu Yang, Ping Yu

**Affiliations:** Department of Intensive Care Unit, Wuxi Second People's Hospital, Wuxi, Jiangsu, China; Middle Technical University, IRAQ

## Abstract

**Objective:**

To develop evidence-based audit indicators for urinary leakage prevention and management in ICU patients with indwelling catheters, to evaluate current clinical compliance with these indicators, and to analyze barriers and facilitators for evidence implementation using the JBI Clinical Evidence Application Model as a theoretical framework.

**Methods:**

Using the Clinical Evidence Application Model from the Joanna Briggs Institute(JBI)Centre for Evidence in Healthcare in Australia as the theoretical framework,clinical nursing issues were identified.Literature searches were conducted,evidence was evaluated and synthesised,and review criteria and methods were established.Using convenience sampling,39 nurses and 75 indwelling catheterised patients in the General ICU(North Campus) of Wuxi Second People's Hospital, Wuxi, Jiangsu Province, China were selected for baseline review between April and May 2025.Barriers were analysed and change strategies developed based on baseline findings.

**Results:**

A total of 22 pieces of evidence on urinary leakage prevention and management for ICU patients with indwelling urinary catheters were introduced,leading to the formulation of 21 review indicators. And among those,16 showed that compliance rate was less than 60%. There were 27 barriers and 17 facilitators found by analysis. 25 change strategies were made according to this.

**Conclusion:**

There are big differences between what we know from research about how to stop or deal with urine leaking in intensive care patients who have a tube in their bladder and what doctors and nurses do in hospitals right now. We need special sets of rules called “evidence-based practice protocols” that use tricks from studies to help doctors and nurses give better care to sick people in the hospital.

## 1. Introduction

Urinary leakage is the most common non-infectious complication,occurring at a rate of 10.6%in short-term catheterization and 52.1%in long-term patients [1]. ICU patient is critically ill, so it’s necessary to precisely monitor urine output by routine observation. To ease physical and mental discomfort for people who have urinary incontinence or retention,indwelling catheterization is frequently used.Long term catheterization will cause many different problems which seriously affect the quality of patients'life.Surveys show [2,3] That catheter leakage happens in 20% to 30% of cases. Leakage results in macerated perineal skin and higher chances of pressure injury. It could lead to CAUTI, longer hospital stays and more healthcare cost. According to research [4] That is, patients who have leakage have 2.5 times the risk of CAUTI than those without leakage.Furthermore, the frequent catheter changes needed because of leakage cause more damage to the urethra mechanically, making a bad circle. Despite there being clear guidelines on catheter management from both domestic and foreign experts [5,6], There’s still quite a gap between what we do clinically and what is best based on evidence.Surveys show [7] only 35% of ICU nurses stick to standard catheter size and balloon inflation procedures.Leakage is mostly handled with guesswork (such as pressure setting), without specific tests and treatment plans.The special ICU environment with patients who can’t stop moving around, constant changing positions, and lots of different tubes at once makes it much harder to handle leaks. In light of these points, we adopt the JBI Center for Evidence-based Healthcare in Australia’s Clinical Evidence Application Model from the Joanna Briggs Institute (JBI) as our theoretical framework.A multidisciplinary team was formed to apply evidence-based methods in identifying optimal evidence for preventing and managing urinary leakage in indwelling catheter patients.Review criteria were established to identify barriers to applying best evidence,and action strategies were formulated to facilitate the translation of evidence into clinical practice.

## 2. Materials and methods

### 2.1. Build a team

Form a multidisciplinary evidence-based practice team comprising 12 members, including experts in clinical management,specialized nursing,and evidence-based support.Invite one senior researcher from Fudan University's Evidence-Based Nursing Center to serve as the project's chief consultant,providing guidance on evidence-based methodology,overseeing protocol design,and ensuring quality control throughout the process; Co-lead the initiative with one Deputy Director of Nursing and one ICU Department Director to coordinate medical decision integration,process standardization,and cross-departmental resource coordination,ensuring decision management.Operational coordination was entrusted to a Head Nurse, who served as Team Leader and was responsible for strategic project planning and timeline governance. Two ICU Ward Nurse Managers were appointed as Deputies to oversee the standardisation of competencies, implementation of protocols, and quality monitoring. The main implementation team consisted of four clinical nurse specialists who were purposefully selected from different backgrounds. Two had formal certification in EBP, and another had 10 years’ worth of urological experience. They were supposed to take these guidelines and turn them into real interventions, then lead the change in practice. To guarantee smooth patient management, attending doctors from Urology and Intensive Care Medicine were included in the team so that both therapeutic and nursing approaches would be consistent right from the start.A nursing postgraduate student was responsible for evidence acquisition,the development of audit criteria,and barrier factor analysis.

### 2.2. Identify evidence-based nursing problems

Based on an analysis of the current practices for preventing and managing urinary leakage in patients with indwelling catheters in the ICU of a tertiary hospital in Wuxi,Jiangsu Province,China,several gaps between clinical practice and evidence-based standards were identified.These include a lack of standardized assessment during catheterization,fragmented interventions with poor coordination across key aspects such as catheter size selection,fixation methods,and balloon management,and weak collaborative decision-making between medical and nursing staff.Additionally,knowledge regarding urinary leakage management is insufficient,and preventive measures against catheter blockage are inadequate.To address these gaps,this study aims to establish an evidence-based practice protocol for the prevention and management of urinary leakage in ICU patients with indwelling catheters.

All the staff signed the informed consent.This study was approved by the Ethics Committee of Wuxi Second People's Hospital(No.2024-Y341).The study was conducted in accordance with the Declaration of Helsinki(as revised in 2013).

### 2.3. Literature retrieval

Based on the PIPOST Model from the JBI Centre for Evidence-Based Healthcare [8],identify evidence-based nursing questions. Conduct searches top-down according to the 6S Pyramid Model,constructing search terms by combining subject headings and free-text keywords. The search timeframe spans from January 2014 to December 30,2024. Systematically searched relevant databases:UpToDate,BMJ Best Practice,the Joanna Briggs Institute(JBI)Evidence-Based Healthcare Centre database,Cochrane Library,PubMed,Embase,Web of Science,the National Institute for Health and Care Excellence(NICE),the Registered Nurses'Association of Ontario(RNAO),the National Guideline Clearinghouse(NGC),MedlinePlus,the American Urological Association(AUA),and the European Association of Urology(EAU).UpToDate,JBI Centre for Evidence-Based Healthcare,Cochrane Library,RNAO,Scottish Intercollegiate Guidelines Network,International Guidelines Network,NICE Guidelines Library,New Zealand Guidelines Group,Critical Care Specialty Committee,Diabetes Specialty Committee,China Guidelines Network,Dutch Medical Abstracts Database,PubMed,China Biomedical Literature Database,China National Knowledge Infrastructure(CNKI),etc. The following Chinese search keywords were used:“Critical illness/Severe illness/ICU(Intensive Care Unit)/ Intensive Care Unit”,“Urinary catheter/Catheter/Indwelling urinary catheterization/Urinary catheter insertion”,“Urine leakage/Leakage/”.The following English search keywords were used:ICU/Critical illness*/critically ill/Critical care/Intensive Care unit”“Indwelling catheterization/Indwelling urinary catheterization/Urinary catheter/Catheterization/Urinary catheter insertion”“Urinary leakage management/Leakage control.” A total of 8 articles were ultimately included [9–16]. Among these, 2 were clinical decision aids [9,10], 2 were guidelines [11,12], 1 was an expert consensus [13], and 3 were randomized controlled trials [14–16].

### 2.4. Evaluate and summarize the best evidence

**(1) Literature Quality Assessment.**Two evaluators trained in evidence-based practice independently assessed the guidelines using the Appraisal of Guidelines for Research and Evaluation(AGREE II)tool [17].Expert consensus statements were evaluated according to the JBI Center for Evidence-Based Healthcare criteria [18].Clinical decision aids and evidence summaries were assessed using tools appropriate to their evidence type [19].In cases of disagreement, third-party adjudication is sought.**(2)Evidence item evaluation.**During evidence item extraction,researchers systematically collate evidence items from included literature.When evidence conflicts arise,strict adherence to a four-tier screening principle ensures evidence authority and applicability:evidence-based evidence takes precedence,high-quality evidence takes precedence,most recently published evidence takes precedence,and domestic guidelines take precedence.The JBI Evidence Recommendation Level System(2014 Edition)is applied to determine the recommendation level for included evidence.The evidence-based practice team convenes group meetings involving five stakeholders:one ICU attending physician,one senior ICU nurse with 20 years of experience,one head of the nursing department's catheterization team,one urologist,and one ICU patient who has undergone catheter removal and is in the recovery phase with sufficient consciousness for communication.Recommendations are made based on the feasibility,appropriateness,clinical significance,and effectiveness of evidence application,as well as the clinical implications and effectiveness of evidence translation.One urologist and one ICU patient who had undergone catheter removal and was in the recovery phase with sufficient consciousness to communicate.Based on the feasibility,appropriateness,clinical significance,and effectiveness of evidence application,combined with the clinical context of evidence translation and ICU resource allocation,evidence is scored using a 1–10 point scale.

### 2.5. Review criteria development and baseline assessment

Guided by principles of scientific rigor, credibility, effectiveness, and the evidence's measurability, the evidence-based team convened core members to discuss and develop 21 review criteria based on 22 best-evidence items. Review criterion compliance rate = Number of practitioners adhering to the criteria / Total number of practitioners surveyed × 100%.Recruitment Period and Informed Consent:April 1, 2025 – May 31, 2025: Using convenience sampling, 39 nurses and 75 indwelling catheter patients from the General ICU (North Campus) of a tertiary hospital in Wuxi were selected as baseline review subjects. All participants provided written informed consent to voluntarily engage in this study.Patient Inclusion Criteria: Currently indwelling catheterized; age ≥ 18 years; voluntary participation with written informed consent.Patient Exclusion Criteria: Critically ill patients with an anticipated hospital stay < 24 hours; those transferred or deceased during the study period; those unable to cooperate due to illness or other reasons.Nurse Inclusion Criteria: Possession of a valid nursing license; ≥ 1 year of ICU work experience; voluntary participation with written informed consent.Nurse Exclusion Criteria: Nurses on advanced training, rotation, internship, or maternity/paternity leave.Based on the included best evidence, theSurvey Questionnaire on Evidence Awareness for Urine Leakage Prevention and Management in ICU Patients with Indwelling Catheters was independently designed for nurse review. Five experts evaluated the questionnaire's content validity index (CVI), yielding a CVI of 0.822.

### 2.6. Barrier analysis and action strategy development

For review indicators with compliance rates<60%,the Integrated Action Promotion Framework for Research Implementation in Health Services (i-PARIHS) [20]was applied.Focusing on three dimensions—innovation(I), recipient(R),and context(C)—barriers were.

### 2.7. Statistical methods

Data analysis was performed using SPSS 19.0.Normally distributed quantitative data are presented as mean±standard deviation(x ± s);categorical data are expressed as frequency and percentage(%).

## 3. Results

### 3.1. Included evidence

Initial searches yielded 25 evidence items.Three were excluded due to poor feasibility and clinical applicability:"Recommendation of male standard-length catheters for specific female patient groups,”“Prohibition of female-length catheters for male catheterization,"and"Control of balloon filling volume and catheter curvature."Consequently,22 best evidence items were synthesized across five domains:catheter selection,balloon management,urinary catheter obstruction prevention,medication strategies,and personnel training(**Table 1**).

**Table 1:** Evidence, review indicators, review subjects, and results for urine leakage prevention and management in ICU patients with indwelling urinary.

Evidence dimension	Content of evidence	Review indicator	Review object	Review method	Review result(Clinicalcompliancerate (%)
**Catheter Selection**	1. Unless otherwise clinically indicated,use the smallest possible drainage catheter to minimize irritation and trauma to the bladder neck and urethra.	1. Healthcare providers jointly assess indications for indwelling catheterization,prioritizing smaller catheter sizes.	Patients (n = 75)	On-site checking	0
	2. To prevent urethral injury,long-term use of 16-gauge or larger catheters is not recommended.	2. The responsible nurse should assess the necessity of using size 16 or larger catheters daily.	Patients (n = 75)	On-site checking	0
	3. For patients requiring long-term catheterization,silicone catheter material is selected.	3. For patients requiring long-term indwelling catheters,nurses should select silicone-based catheter materials.	Patients (n = 75)	On-site checking	0
	4. For clear urine without debris,grit,or hematuria,select a 12–14F catheter;for smaller adults,a 10F catheter may be chosen.For mildly cloudy urine with or without small clots,no or mild sediment,and no or mild debris,select a 16F catheter.For moderate to heavy sediment,fragments,or hematuria with moderate clots,select an 18F catheter.For severe hematuria or patients requiring irrigation,select a 20–24F catheter.	4. Healthcare providers should jointly assess the patient's urinary symptoms to select an appropriate catheter.	Patients (n = 75)	On-site checking	0
	5. Latex-based catheters are reserved for patients without latex allergies and for short-term use(<14 days).	5. Upon admission,nurses assess patients for latex allergies	Patients (n = 75)	Check nursing records	0
	6. When urinary leakage occurs with a long-term indwelling catheter,replace it with a new catheter that is 2F to 4F larger in size than the previous one.	6. When urinary leakage occurs with a long-term indwelling catheter,the responsible nurse should replace it with a new catheter that is 2F to 4F larger than the previous size.	Patients (n = 21)	On-site checking	0
**Balloon Management**	7. For different types of urinary catheters,inflate the balloon according to the manufacturer's instructions to avoid underfilling or overfilling.	7. Before the responsible nurse performs the procedure,conduct a double-check with the manufacturer's instructions to confirm the required balloon filling volume.After catheter insertion,fully document the filling process.	Nurses (n = 39)	Documents,Nursing Records	0
	8. It is not recommended to instill more than 10 ml of fluid into the balloon of a long-term indwelling urinary catheter.	8. The responsible nurse shall assess patients with long-term indwelling urinary catheters daily,ensuring balloon irrigation does not exceed 10 ml.	Nurses (n = 39)	Check nursing records	0
	9. Performing three pre-inflation cycles of the catheter balloon prior to insertion, ensuring uniform inflation followed by complete deflation, effectively reduces urinary leakage.	9. The responsible nurse should perform three checks of the balloon before catheter insertion.	Nurses (n = 39)	On-site questioning	28.2
**Preventing blockage**	10. Daily urine output exceeds 2000ml.	10. Responsible nurse records urine output per shift.	Patients (n = 75)	Check nursing records	100
	11. Maintain a balanced diet and implement bowel management to prevent constipation.	11. The responsible nurse shall record the patient's fluid intake and output during each shift and evaluate bowel movements.	Patients (n = 75)	Check nursing records	100
	12. Empty the urinary catheter bag regularly and ensure the catheter is free of kinks to minimize mechanical blockages.	12. Empty the urinary catheter bag during each shift,check for kinks in the catheter tube,and minimize mechanical blockages.	Patients (n = 75)	On-site checking	90.7
	13. If the drainage bag is positioned too low,urine in the drainage tube may draw mucus into the catheter pores due to the siphon effect.Therefore,ensure the drainage bag is elevated.	13. Nurses correctly position the drainage bag.	Patients (n = 75)	On-site checking	90.7
	14. Develop a scientifically sound and reasonable bladder irrigation protocol or urinary catheter maintenance plan.	14. The department has a scientifically sound and reasonable bladder irrigation protocol or urinary catheter maintenance protocol.	System	Review documents	0
	15. For recurrent obstructions,rule out the possibility of bladder stones through appropriate examinations.	15. For patients experiencing recurrent blockages,the responsible nurse is aware of the need to rule out the possibility of bladder stones.	Nurses (n = 39)	On-site checking	0
	16. Reduce bacterial colonization by using closed catheter systems and regularly replacing catheters to minimize floc formation.	16. The responsible nurse assesses the integrity of the closed catheter system during each shift.	Nurses (n = 39)	On-site checking	100
		17. The department has established protocols and standard operating procedures(SOPs)for regular catheter replacement.	System	Document Review	0
**Pharmacotherapy**	17. Anticholinergic medications may be used to reduce catheter-related pain and leakage.	18. The responsible nurse understands the effects of medications on catheter-related pain,spasms,and leakage.	Nurses (n = 39)	Questionnaire Survey	0
	18. Intravesical botulinum toxin may be used for severe catheter spasm and leakage.				
	19. Urinary leakage caused by detrusor overactivity/uninhibited bladder contractions can be managed by reducing balloon volume or using anticholinergic medications.				
**Staff Training**	20. Healthcare professionals involved in catheter management require comprehensive knowledge of appropriate catheter products and related equipment.	19. Departments should maintain systematic training and assessment records for catheter products and equipment knowledge. Training and assessment records	System	Document Review	0
	21. Understanding catheterization techniques and routine catheter care helps reduce leakage caused by mechanical obstruction.	20. The department has established nursing protocols for managing and preventing urinary leakage related to catheterization techniques and catheter care.	System	Review documents	0
	22. Under conditions ensuring minimal catheter tension,catheters for both male and female patients may be secured to the abdomen or thigh.	21. Responsible nurse ties off the catheter as per the procedure without much tension on the catheter.	Patients (n = 75)	On-site checking	29.3

### 3.2. Baseline evaluation results

This study established 21 review indicators,grouped as follows based on compliance:3 indicators(10,11,16)achieved 100%compliance;14 indicators (1,2,3,4,5,6,7,8,14,15,17,18,19,20)had a compliance rate of 0%;2 indicators(9,21) showed compliance rates below 60%;and the remaining 2 indicators(12,13)had compliance rates above 60%.Table 1 presents the evidence included for urinary leakage prevention and management in ICU patients with indwelling urinary catheters,along with the corresponding review indicators,review methods,and results.The low compliance rates across multiple indicators reveal substantial gaps between current clinical practice and best evidence. These gaps increase the risk of urethral injury, catheter-related infection, leakage, pressure injury, and prolonged hospital stay. Improving adherence to evidence-based protocols is critical to enhancing patient safety and care quality in the ICU.

### 3.3. Analysis of barriers and facilitators

Based on the baseline audit results, a multi-discipline team consisting of a department manager, 2 critical care CNS, urologist, and 2 EBP specialists carried out structured brainstorming sessions to find out how to implement it. Using i-PARIHS framework, the team looked at what affected adopting it; they checked if the evidence fit and was possible, if nurses and patients liked it, and about the place, having enough things and people working well together. These talks went round and round, helping pick out big obstacles and work on plans just for them,as shown in Table 2.

**Table 2:** Barriers, facilitators, and action strategies for urinary leakage prevention and management in ICU patients with indwelling catheters.

Reviewindicator	Barriers	Facilitating factors	Action strategies
Review Indicators 1,2,4,5,7,8,9,15,18,21	1. Healthcare providers lack awareness of the best evidence and have unclear understanding of the advantages of smaller-gauge urinary catheters(e.g.,reduced risk of urethral injury and infection). 2. During nursing rounds,the absence of standardized assessment tools or protocols for evaluating indwelling catheter placement renders joint assessments largely perfunctory. 3. Junior nurses constitute 66.9%of the workforce,lacking specialized experience and insufficient knowledge in preventing and managing leakage in indwelling catheter patients. 4. Physicians dominate catheter selection,frequently disregarding nurses'recommendations. 5. Assessment forms lack specific content,and evidence has not been translated into accessible,actionable formats. 6. There is no standardized urinary symptom assessment scale or catheter selection criteria. 7. Integration of nursing and medical handover content is lacking,preventing effective transmission of nursing assessment results to the medical team. 8.Latex allergy is not included in departmental admission assessment forms. 9.Communication with family members revealed minimal awareness of latex allergy sources and related knowledge. 10. Evidence requires modification of existing nursing practices;current protocols lack a step for nurses to verify instructions. 11. In balloon catheter management,evidence descriptions are unclear,and the filling process documentation is cumbersome,failing to demonstrate direct nursing value.Nursing documentation standards and requirements must be discussed with the documentation team. 12. Nurses perceive"empirical fluid administration”(uniform 10 ml for both male and female patients)as more efficient,with insufficient recognition of the clinical benefits of precise fluid administration. 13. Difficulties in implementing evidence exist,as there are no simple measurement tools available for monitoring already-inserted urinary catheter balloons. 14. Increased workload has dampened nurses'enthusiasm for practice changes. 15. Departmental nursing protocols have not been updated based on recent evidence,with balloon checks performed only once prior to procedures. 16. Inertia persists,with some nurses relying on visual checks during shift handover instead of standardized assessments.	1. The Nursing Department has prioritized catheter-related infection prevention as a key quality improvement initiative this year,with high attention. 2. Surveys indicate most nurses are willing to learn knowledge related to preventing and managing urinary leakage from indwelling catheters. 3. Three-quarters of the department's nurses hold bachelor's degrees,indicating strong knowledge absorption capacity. 4.Medical and nursing staff frequently organize multidisciplinary case conferences to showcase successful precision catheter selection cases. 5. National nursing quality improvement initiatives mandate enhanced medication safety management capabilities. 6. With rapid advancements in AI technology in China,personalized medication risk profiles and knowledge graphs can be developed.Department staff demonstrate strong willingness to learn new technologies. 7. The department includes core members of the Nursing Department's Catheterization Team,who conduct regular oversight.	1. Medical and nursing teams share aligned communication goals,with catheter selection based on prescribed specifications and materials. 2. Based on best evidence,the Standardized Process for Catheter Selection and Retention Assessment was developed,clarifying responsibilities for both parties and embedded in the electronic medical record. 3. Implemented a"four-eyes principle"assessment process:-Initial nursing assessment(symptom collection+specimen submission)-Joint physician-nurse review(review test results+confirm catheter selection)-Dual signature confirmation. 4. Implement mandatory assessment fields in the critical care"Jiahe"EMR system,linked to a catheter model knowledge base. 5. Implement a"Catheter Safety Morning Meeting”:Incorporate urinary catheter leakage data into the daily morning handover. 6. Utilize departmental quality control boards to display monthly catheter maintenance metrics(e.g.,leakage incidence trend charts). 7. Add a"Tiered Catheter Management Training Program”:Reinforce foundational procedures(e.g.,proper balloon inflation)for junior nurses,while providing advanced training on complex scenarios(e.g.,drug-related leakage)for senior nurses. 8. Develop a"Catheter Safety Passport”:A pictorial self-assessment guide(covering risk identification such as leakage and loose fixation). 9. Have departmental"skilled practitioners"create instructional videos on preventing and managing urinary leakage in indwelling catheter patients.Convert these into QR code-linked tutorials(including balloon management precautions)and share them in departmental work groups for on-demand learning. 10. For patients with indwelling catheters exceeding 48 hours,revise the SBAR handover form to include catheter placement reminders,ensuring catheter safety and leakage prevention during retention. 11.Embed a"Catheter Maintenance Assistant"in the department's mobile nursing terminals to provide real-time procedural guidance(e.g.,animated demonstrations for balloon inflation). 12. Automatically trigger consultation requests when a patient experiences recurrent leakage within three days.
**Review indicators 14,17,19–20**	17. Existing SOPs lack clear differentiation in replacement cycles for various catheter types(e.g.,silicone,latex),resulting in ambiguous operational standards for nurses. 18. Content complexity and update delays, coupled with rapid catheter product iterations and insufficient training material refresh rates, result in outdated knowledge. 19. Evidence primarily originates from international sources, lacking localised experience. 20. Critically ill patients often present with complex and physiologically altered genitourinary anatomy, complicating catheter placement. Current training remains essentially didactic and lacks high-fidelity simulation, leaving nurses underprepared to manage anatomical variations—such as the heightened vulnerability of the male urethral isthmus to iatrogenic injury. 21. Implementing evidence-based catheter management requires coordination across several clinical departments and specialities, introducing substantial operational and interdisciplinary challenges. 22. Operational complexity arising from a lack of quantifiable benchmarks.	8. The critical care"Jiahe"system is currently undergoing testing,with engineers stationed within the department to enhance knowledge acquisition modules.2.Nurses demonstrate high acceptance of the tools. 9. The hospital has a dedicated catheter care team with relevant operational protocols;the urinary leakage assessment process and its details can be revised and refined. 10. Infection control requirements necessitate training on standardized catheter usage. 11. Departments routinely use platforms like Tencent Meeting and DingTalk to integrate training attendance,assessments,and record-keeping. 12. The Nursing Department has newly revised training systems for all nursing levels this year,developing modular courses.Course design is primarily led by the department's specialized teams,with the catheterization team responsible for training all hospital nurses. 13. Patient safety goals include urinary catheter-related complications as core monitoring targets. 14. The department was recognized as an outstanding teaching team last year and possesses extensive instructional experience.	13. Implemented a"micro-learning"model within the department(each session≤15 minutes),focusing on single skill points in preventing and managing urinary leakage from indwelling catheters,with assessments to ensure mastery by all. 14. Senior nurses serve as"clinical mentors,"conducting bedside teaching using case studies. 15. Formed a catheter quality control team comprising urologists,critical care staff,and infection control personnel,with clearly defined responsibilities. 16. Strengthen the responsibilities of the catheter quality control team and establish quality control standards for preventing and managing urinary leakage in ICU patients with indwelling urinary catheters.The department's lead nurse will conduct daily inspections,identify issues,and incorporate findings into performance evaluations. 17. Monthly selection of representative leakage cases for critical care nurses to reverse-engineer treatment deficiencies.
Review Indicators 3,6	23.The department's routine supply of catheter sizes is limited,and the procurement process prioritizes cost over clinical needs,failing to meet individualized requirements. 24.Nurses empirically select original catheter models or increase size by only 1F. 25.Lack of standardised protocols for individualised adjustments. Evidence supporting 2F upgrades for leakage or 4F upgrades for recurrent leakage remains underutilised,hindering nurse implementation. 26. Existing standardised protocols do not incorporate structured guidance for individualised catheter adjustment, particularly in implementing evidence-based sizing strategies. 27. Some younger nurses exhibit poor communication skills and risk-averse tendencies,fearing catheter upgrade may lead to placement difficulties or patient complaints.	15. The department operates a"Micro-U Classroom,"where senior healthcare providers engage with families during daily rounds,including disease-related health education. 16. The head of the Nursing Department's Evidence-Based Practice Team is based in this department and has experience successfully translating evidence-based projects.3.The Nursing Department has incentive mechanisms for evidence translation projects. 17. The department is undergoing re-evaluation for its Grade III A-level and provincial key hospital status,with all record-keeping standards currently being updated.	18. Inventory is dynamically adjusted through enhanced coordination with the procurement center to ensure immediate availability of all catheter models for distribution or interdepartmental borrowing. 19.Senior nurses demonstrate standardized operating procedures. 20.Specialized training plans include professional development on preventing and managing urinary leakage in ICU patients with indwelling catheters,nursing rounds,and emergency response drills. 21. Organise practical workshops using urethral pressure models to visually demonstrate the differential mechanical stress exerted by various catheter types on urethral tissue. 22. Collaborate with the hospital IT department to integrate a “medication-nursing” smart alert module into the electronic medical record system, triggering automated reminders for catheter assessment when specific medications are prescribed. 23.Update selection criteria for silicone vs.latex catheters,create mnemonics to aid healthcare providers in clinical decision-making. 24. Create a structured patient education protocol for ICU settings, including visual aids and QR-accessible micro-videos, to be delivered during daily nursing rounds based on patient consciousness levels 25. Implement a structured family education program delivered by senior nurses during designated visiting hours, focusing on catheter management principles and early leakage recognition, supported by illustrated reference cards.

## 4. Discussion

### 4.1. Development of evidence-based audit criteria for the prevention and management of catheter-associated urinary leakage in ICU patients

Scientific audit criteria should be applied so as to close the evidencetopractice gap for managing catheterassociatedurinary leakage in the ICU, which is a clinically relevant problem that can negatively affect patient comfort, increase the risk of infection, and lead to longer hospital stays [21,22].And those kinds of indicators have to take into account the best available evidence along with what’s feasible clinically and among professionals too [21]. Based on the JBI Evidence Translation Framework, we formed a panel consisting of nursing leaders, ICU clinicians, and urological experts. After the panel performed a systematic search and synthesis of all the available evidence, they used the FAME principles to generate 21 audit criteria. These criteria cover five major areas: catheter choice, balloon handling, obstruction avoidance, drug use, and staff education, creating a useful way to check if people follow good nursing ideas based on facts. To make sure everything was the same, all the people looking at things got the same training about how to do it before they started checking at the beginning.

### 4.2. Conduct standardized clinical reviews of urinary leakage prevention and management for ICU patients with indwelling urinary catheters to provide a basis for analyzing barriers

Successfully carrying out Evidence Based Care depends on correctly finding the differences between what we know from evidence and how nurses actually do things. Our audit shows there’s a big difference between what’s happening now and the best evidence for stopping and dealing with urine leaking out in ICUs when people have a tube inside their bladder all the time. Out of 21 indicators checked, only 3 reached full compliance; 14 had none. In total, 16 indicators showed compliance rates under 60%, which means there are many chances to get better at doing things right, especially when it comes to choosing catheters, handling balloons, and teaching staff. (1) Systemic Level: There’s no department with its own group, procedures, standardised methods, unified urinary symptom evaluation scales, or criteria for choosing catheters to prevent and handle leakage. This takes away clear directions from nursing workers when it comes to checking on and choosing catheters. Therefore, before translating clinical evidence, multidisciplinary teams need to establish organisational structures. This could be done in different ways by using the projects teams previous evidence based practice such as [23]such as online meetings,video education [24], Hands-on demonstrations,small group discussion.(2) Urinary Catheter Selection: Audit indicators 1–6 all have 0% compliance rate, which shows that there’s no uniform assessment tool and procedure for urinary catheter selection among health care providers, also indicating a lack of knowledge about best evidence. Field interview results showed that most healthcare workers had a vague idea about how smaller catheters can reduce the risk of urethral injuries and infections. Moreover, it was found that doctors chose the catheters and did not take nurses’ suggestions into consideration.In practical operations,nursing staff mostly relied on empirical selection rather than evidence-based decision-making. (3) Balloon Management:The compliance rate for Audit Indicators 7–9 ranged from 0%to 28.2%.Interviews with 10 nurses at the N2 level or above showed that 8 of them considered"empirical inflation"more efficient,with insufficient clear awareness of the clinical benefits of accurate balloon inflation.Additionally,the current nursing protocols in the department had not been updated in a timely manner based on the latest evidence,indicating that operational standards for balloon management had not been effectively implemented.In the future,the department could simplify operational procedures and reduce unnecessary documentation in line with evidence. Furthermore,case-based teaching and bedside demonstrations could be adopted to enhance nurses'recognition of and enthusiasm for evidence implementation [25–26].(4) Clogging Prevention:The compliance rate for Audit Indicators 14–15 was 0%,indicating that the department lacks scientifically and rationally developed bladder irrigation protocols or urinary catheter maintenance plans.Additionally,nurses were unfamiliar with the management procedures for patients with recurrent catheter clogging. Based on this, the next round of improvements could involve setting up standardized bladder irrigation procedures at a minimum, and also enhancing nurses’ training in skills related to dealing with patients who have recurrent clog issues so as to enable them to spot possible dangers such as bladder stones right away [27].(5) Staff training: The compliance rate for audit indicators 19–21 were between 0%−29.3%. Survey results showed that there was no sufficient departmental training about catheter management, a knowledge gap among nursing staff and also there is a need for improvement in terms of the format and content of the current training program. Current training mainly depends on lectures with few opportunities for high-fidelity simulation exercises which makes it hard for nurses to form a detailed picture of how urethra shapes differ from person to person. So to close those gaps we would recommend adding in high fidelity simulations as well as workshops [28] into the training framework to improve the transfer of operational skills. At the same time, it is necessary to establish a dynamic update mechanism for training content so that clinical practitioners can learn the latest knowledge and procedural standards. This will help them understand and use the best evidence for preventing and treating urinary leakage in ICU patients with catheters better. Currently, studies on how to prevent and deal with urinary leakage in ICU patients who have catheters mostly focus on finding out which things affect it and making plans to change those things. But when it comes to standardized management protocol studies, there aren’t as many. Clinical healthcare teams might do some surveys about how well they’re taking care of people with catheters who have urine leaking, to see what’s happening right now with stopping the leak and dealing with it. Based on these findings, they would then come up with a plan that involves different kinds of people working together to take care of the problem and put into action ways to make sure everything is done properly and all at once. This way can make the occurrence of urinary leakage in ICU patients with indwelling catheters less likely and lower the chance of getting an infection from using a catheter.

## 5. Conclusion

Urinary leakage management audits within our ICU show that clinical practices tend to be largely reactive and based on individual experience instead of standardized protocols. It’s due to two main reasons: one is that when applying international evidence directly, it doesn’t consider local staffing models and resources; another reason is that there aren’t any culturally appropriate patient assessment tools. In moving forward, we need to get past just bringing in guidelines and start co-designing practical interventions with the frontline teams. First thing first, we need to establish a single procedure framework–starting off with a validated risk-assessment scale and a clear decision-making algorithm for nurses. We expect such practice standardization can not only lower leakage occurrences but also lessen related problems, so as to set up a fresh criterion for evidence-based catheter care within our particular clinical setting.
