# Supplementary material for: Implementing evidence-based practices for urinary leakage prevention in ICU patients with indwelling catheters: A JBI-guided baseline review
Source: PLoS One. 2026 Jul 10;21(7):e0351406. doi: 10.1371/journal.pone.0351406 (PMC13354004; doi:10.1371/journal.pone.0351406)
Supplement: Attachment — Submitted filename: 1.Response to Reviewers.docx [file pone.0351406.s002.docx]

# **Response to Reviewers**

To: The Editor
And
To: The Reviewers

We wish to express our sincere gratitude to you and the reviewers for the time and effort dedicated to reviewing our manuscript entitled “**Development of Review Indicators and Analysis of Barriers for Urine Leakage Prevention and Management in ICU Patients with Indwelling Urinary Catheter”** (Manuscript ID: PONE-D-25-60399). We deeply appreciate the insightful comments and constructive suggestions provided by the reviewers. These comments have been invaluable in helping us to significantly improve the quality and clarity of our work. We have carefully considered all the points raised and have made extensive revisions to the manuscript accordingly. In this point-by-point response, we have addressed each comment in detail.

Following the reviewer’s suggestion, we have revised the manuscript title to: Implementing evidence-based practice for urine leakage prevention in ICU patients with indwelling urinary catheters:A JBI-guided baseline review.

Response to the Academic Editor

Comment 1(Journal Requirement #1):Please ensure that your manuscript meets PLOS ONE's style requirements, including those for file naming.

Response 1:We have carefully reviewed the PLOS ONE style templates and reformatted our manuscript accordingly. The title page, headings, line spacing, margins, and file naming now fully comply with the journal's requirements.

Comment 2(Journal Requirement #2):PLOS requires an ORCID iD for the corresponding author. Please ensure that you have an ORCID iD and that it is validated in Editorial Manager.

Response 2:The corresponding author (Ping Yu) has verified their ORCID iD in Editorial Manager using the Fetch/Validate link. The ORCID iD is now validated and will appear in the published article.

Comment 3(Journal Requirement #3):Your ethics statement should only appear in the Methods section of your manuscript.

Response3:We have confirmed that the ethics statement appears only in the Methods section (Section 2.2). All informed consent, ethics approval, and declaration of Helsinki statements have been moved to the end of Section 2.2. No ethics statement appears elsewhere in the manuscript.

Comment 4 (Journal Requirement #4 & #5):Please provide a complete Data Availability Statement.

Response4:We have completed the Data Availability Statement in the submission system as follows:The data underlying this study contain sensitive patient information and cannot be made publicly available due to ethical restrictions imposed by the Ethics Committee of Wuxi Second People's Hospital (Approval No. 2024-Y341). Requests for data access should be directed to the Ethics Committee of Wuxi Second People's Hospital (Email: wxeyzzjd@163.com; Tel: +86 510 66618534).

Comment 5(Journal Requirement #6):Please update your submission to use the PLOS LaTeX template.

Response5:We have reformatted the manuscript using the PLOS LaTeX template. The revised manuscript has been compiled accordingly and is now being submitted in compliance with this requirement.

Comment 6(Journal Requirement #7):Please amend your authorship list in your manuscript file to include author Ping Yu, Hong Bian, Jingjing Yan, Zhiyin Zhou, Zhengyu Yang.

Response6:We have added the complete authorship list with the correct order and designated corresponding author to the title page of the revised manuscript, as requested.

Comment 7(Journal Requirement #8) :Please upload a copy of Figure 1, to which you refer in your text on page 5. If the figure is no longer to be included, please remove all reference to it within the text.

Response7:We have removed Figure 1 from the submission. All references to Figure 1 have been deleted from the text (Section 2.3). The search strategy is now fully described in the text, which is sufficient for readers to reproduce the search.

Comment 8 (Journal Requirement #9):If the reviewer comments include a recommendation to cite specific previously published works, please review and evaluate these publications.

Response8:The reviewer did not recommend any specific previously published works for citation. Therefore, no changes were made to the reference list on this point.

Comment 9 (Journal Requirement #10):Please review your reference list to ensure that it is complete and correct. If you have cited papers that have been retracted, please include the rationale for doing so.

Response9:The entire reference list has been rechecked, updated, and standardized to fully meet PLOS ONE Vancouver style requirements. No retracted articles are included in the references.

## Response to Reviewer

## Reviewer Comment 1:Suggest change title into: "Implementing evidence-based practices for urinary leakage prevention in ICU patients: A JBI-guided baseline review."

Response:We thank the reviewer for this constructive suggestion. We have revised the title accordingly. The new title is:Implementing evidence-based practices for urinary leakage prevention in ICU patients with indwelling catheters: A JBI-guided baseline review.

This change has been made on the title page of the revised manuscript.

Reviewer Comment 2:The study's objective is unclear; please rephrase it.

Response:We apologize for the lack of clarity. We have rephrased the Objective section in the Abstract to state the aim more directly. The revised Objective now reads:

Objective:To develop evidence-based audit indicators for urinary leakage prevention and management in ICU patients with indwelling catheters, to evaluate current clinical compliance with these indicators, and to analyze barriers and facilitators for evidence implementation using the JBI Clinical Evidence Application Model as a theoretical framework.

Reviewer Comment 3:The results in numbers must be linked to clinical or organizational meaning.

Response: We agree with the reviewer. We have added brief interpretive statements in the Results section (3.2) to clarify the clinical relevance of the quantitative findings. The following text has been added:

"The low compliance rates across multiple indicators reveal substantial gaps between current clinical practice and best evidence. These gaps increase the risk of urethral injury, catheter-related infection, leakage, pressure injury, and prolonged hospital stay. Improving adherence to evidence-based protocols is critical to enhancing patient safety and care quality in the ICU."

The relevant in-depth analyses are further elaborated in the Discussion section (4.2).

Reviewer Comment 4:Reordering the sources to conform to the PLOS guidelines.

Response:We have carefully checked, rearranged, and reformatted all references to fully comply with PLOS ONE’s Vancouver reference style. Author format, title case, punctuation, DOI, and citation order have been uniformly revised.

We sincerely hope that the revised manuscript now meets the publication standards of PLOS ONE. Thank you again for your professional and valuable guidance.

Sincerely,

The Authors

The Second People's Hospital of Wuxi, Jiangsu, China
