# Supplementary material for: Implementing evidence-based practices for urinary leakage prevention in ICU patients with indwelling catheters: A JBI-guided baseline review
Source: PLoS One. 2026 Jul 10;21(7):e0351406. doi: 10.1371/journal.pone.0351406 (PMC13354004; doi:10.1371/journal.pone.0351406)
Supplement: Peer review history

# Peer review: Decision Letter 0

**DOI:** 10.1371/journal.pone.0351406.r001

**Authors:** Hussam Awayid

29 Apr 2026

PONE-D-25-60399

Development of Review Indicators and Analysis of Barriers for Urine Leakage Prevention and Management in ICU Patients with Indwelling Urinary Catheters

PLOS One

Dear Dr. Yu,

Thank you for submitting your manuscript to PLOS ONE. After careful consideration, we feel that it has merit but does not fully meet PLOS ONE’s publication criteria as it currently stands. Therefore, we invite you to submit a revised version of the manuscript that addresses the points raised during the review process.

Please submit your revised manuscript by Jun 12 2026 11:59PM. If you will need more time than this to complete your revisions, please reply to this message or contact the journal office at plosone@plos.org. When you're ready to submit your revision, log on to https://www.editorialmanager.com/pone/ and select the 'Submissions Needing Revision' folder to locate your manuscript file.

Please include the following items when submitting your revised manuscript:

- A letter that responds to each point raised by the academic editor and reviewer(s). You should upload this letter as a separate file labeled 'Response to Reviewers'.
- A marked-up copy of your manuscript that highlights changes made to the original version. You should upload this as a separate file labeled 'Revised Manuscript with Track Changes'.
- An unmarked version of your revised paper without tracked changes. You should upload this as a separate file labeled 'Manuscript'.

If you would like to make changes to your financial disclosure, please include your updated statement in your cover letter. Guidelines for resubmitting your figure files are available below the reviewer comments at the end of this letter.

If applicable, we recommend that you deposit your laboratory protocols in protocols.io to enhance the reproducibility of your results. Protocols.io assigns your protocol its own identifier (DOI) so that it can be cited independently in the future. For instructions see: https://journals.plos.org/plosone/s/submission-guidelines#loc-laboratory-protocols. Additionally, PLOS ONE offers an option for publishing peer-reviewed Lab Protocol articles, which describe protocols hosted on protocols.io. Read more information on sharing protocols at https://plos.org/protocols?utm_medium=editorial-email&utm_source=authorletters&utm_campaign=protocols.

As the corresponding author, your ORCID iD is verified in the submission system and will appear in the published article. PLOS supports the use of ORCID, and we encourage all coauthors to register for an ORCID iD and use it as well. Please encourage your coauthors to verify their ORCID iD within the submission system before final acceptance, as unverified ORCID iDs will not appear in the published article. *Only*  the individual author can complete the verification step; PLOS staff *cannot*  verify ORCID iDs on behalf of authors.

We look forward to receiving your revised manuscript.

Kind regards,

Hussam sami Awayid, Ph.D.

Academic Editor

PLOS One

Journal Requirements:

When submitting your revision, we need you to address these additional requirements.

1. Please ensure that your manuscript meets PLOS ONE's style requirements, including those for file naming. The PLOS ONE style templates can be found at

https://journals.plos.org/plosone/s/file?id=wjVg/PLOSOne_formatting_sample_main_body.pdf and https://journals.plos.org/plosone/s/file?id=ba62/PLOSOne_formatting_sample_title_authors_affiliations.pdf

2. PLOS requires an ORCID iD for the corresponding author in Editorial Manager on papers submitted after December 6th, 2016. Please ensure that you have an ORCID iD and that it is validated in Editorial Manager. To do this, go to ‘Update my Information’ (in the upper left-hand corner of the main menu), and click on the Fetch/Validate link next to the ORCID field. This will take you to the ORCID site and allow you to create a new iD or authenticate a pre-existing iD in Editorial Manager.

3. Your ethics statement should only appear in the Methods section of your manuscript. If your ethics statement is written in any section besides the Methods, please delete it from any other section.

4. Please provide a complete Data Availability Statement in the submission form, ensuring you include all necessary access information or a reason for why you are unable to make your data freely accessible. If your research concerns only data provided within your submission, please write "All data are in the manuscript and/or supporting information files" as your Data Availability Statement.

5. When completing the data availability statement of the submission form, you indicated that you will make your data available on acceptance. We strongly recommend all authors decide on a data sharing plan before acceptance, as the process can be lengthy and hold up publication timelines. Please note that, though access restrictions are acceptable now, your entire data will need to be made freely accessible if your manuscript is accepted for publication. This policy applies to all data except where public deposition would breach compliance with the protocol approved by your research ethics board. If you are unable to adhere to our open data policy, please kindly revise your statement to explain your reasoning and we will seek the editor's input on an exemption. Please be assured that, once you have provided your new statement, the assessment of your exemption will not hold up the peer review process.

6. Please update your submission to use the PLOS LaTeX template. The template and more information on our requirements for LaTeX submissions can be found at http://journals.plos.org/plosone/s/latex.

7. Please amend your authorship list in your manuscript file to include author Ping Yu, Hong Bian, Jingjing Yan, Zhiyin Zhou, Zhengyu Yang.

8. Please upload a copy of Figure 1, to which you refer in your text on page 5. If the figure is no longer to be included as part of the submission please remove all reference to it within the text.

9. If the reviewer comments include a recommendation to cite specific previously published works, please review and evaluate these publications to determine whether they are relevant and should be cited. There is no requirement to cite these works unless the editor has indicated otherwise.

10. Please review your reference list to ensure that it is complete and correct. If you have cited papers that have been retracted, please include the rationale for doing so in the manuscript text, or remove these references and replace them with relevant current references. Any changes to the reference list should be mentioned in the rebuttal letter that accompanies your revised manuscript. If you need to cite a retracted article, indicate the article’s retracted status in the References list and also include a citation and full reference for the retraction notice.

[Note: HTML markup is below. Please do not edit.]

Reviewers' comments:

Reviewer's Responses to Questions

**Comments to the Author**

1. Is the manuscript technically sound, and do the data support the conclusions?

The manuscript must describe a technically sound piece of scientific research with data that supports the conclusions. Experiments must have been conducted rigorously, with appropriate controls, replication, and sample sizes. The conclusions must be drawn appropriately based on the data presented.

Reviewer #1: Partly

**********

2. Has the statistical analysis been performed appropriately and rigorously?

Reviewer #1: I Don't Know

**********

3. Have the authors made all data underlying the findings in their manuscript fully available?

The PLOS Data policy requires authors to make all data underlying the findings described in their manuscript fully available without restriction, with rare exception (please refer to the Data Availability Statement in the manuscript PDF file). The data should be provided as part of the manuscript or its supporting information, or deposited to a public repository. For example, in addition to summary statistics, the data points behind means, medians and variance measures should be available. If there are restrictions on publicly sharing data—e.g. participant privacy or use of data from a third party—those must be specified.

Reviewer #1: Yes

**********

4. Is the manuscript presented in an intelligible fashion and written in standard English?

PLOS ONE does not copyedit accepted manuscripts, so the language in submitted articles must be clear, correct, and unambiguous. Any typographical or grammatical errors should be corrected at revision, so please note any specific errors here.

Reviewer #1: Yes

**********

5. Review Comments to the Author

Please use the space provided to explain your answers to the questions above. You may also include additional comments for the author, including concerns about dual publication, research ethics, or publication ethics. (Please upload your review as an attachment if it exceeds 20,000 characters)

Reviewer #1: suggest chang tittle in to : mplementing evidence-based practices for urinary leakage prevention in ICU patients: a JBI-guided baseline review.

The study's objective is unclear; please rephrase it.

The results in numbers must be linked to clinical or organizational meaning.

Reordering the sources to conform to the PLOS guidelines.

**********

6. PLOS authors have the option to publish the peer review history of their article (what does this mean?). If published, this will include your full peer review and any attached files.

If you choose “no”, your identity will remain anonymous but your review may still be made public.

**Do you want your identity to be public for this peer review?**  For information about this choice, including consent withdrawal, please see our Privacy Policy.

Reviewer #1: No

**********

[NOTE: If reviewer comments were submitted as an attachment file, they will be attached to this email and accessible via the submission site. Please log into your account, locate the manuscript record, and check for the action link "View Attachments". If this link does not appear, there are no attachment files.]

To ensure your figures meet our technical requirements, please review our figure guidelines: https://journals.plos.org/plosone/s/figures

You may also use PLOS’s free figure tool, NAAS, to help you prepare publication quality figures: https://journals.plos.org/plosone/s/figures#loc-tools-for-figure-preparation.

NAAS will assess whether your figures meet our technical requirements by comparing each figure against our figure specifications.

# Author comment: Author response to Decision Letter 1

**DOI:** 10.1371/journal.pone.0351406.r002

25 May 2026

Response to Reviewers

To: The Editor

And

To: The Reviewers

We wish to express our sincere gratitude to you and the reviewers for the time and effort dedicated to reviewing our manuscript entitled “Development of Review Indicators and Analysis of Barriers for Urine Leakage Prevention and Management in ICU Patients with Indwelling Urinary Catheter” (Manuscript ID: PONE-D-25-60399). We deeply appreciate the insightful comments and constructive suggestions provided by the reviewers. These comments have been invaluable in helping us to significantly improve the quality and clarity of our work. We have carefully considered all the points raised and have made extensive revisions to the manuscript accordingly. In this point-by-point response, we have addressed each comment in detail.

Following the reviewer’s suggestion, we have revised the manuscript title to: Implementing evidence-based practice for urine leakage prevention in ICU patients with indwelling urinary catheters:A JBI-guided baseline review.

Response to the Academic Editor

Comment 1(Journal Requirement #1):Please ensure that your manuscript meets PLOS ONE's style requirements, including those for file naming.

Response 1:We have carefully reviewed the PLOS ONE style templates and reformatted our manuscript accordingly. The title page, headings, line spacing, margins, and file naming now fully comply with the journal's requirements.

Comment 2(Journal Requirement #2):PLOS requires an ORCID iD for the corresponding author. Please ensure that you have an ORCID iD and that it is validated in Editorial Manager.

Response 2:The corresponding author (Ping Yu) has verified their ORCID iD in Editorial Manager using the Fetch/Validate link. The ORCID iD is now validated and will appear in the published article.

Comment 3(Journal Requirement #3):Your ethics statement should only appear in the Methods section of your manuscript.

Response3:We have confirmed that the ethics statement appears only in the Methods section (Section 2.2). All informed consent, ethics approval, and declaration of Helsinki statements have been moved to the end of Section 2.2. No ethics statement appears elsewhere in the manuscript.

Comment 4 (Journal Requirement #4 & #5):Please provide a complete Data Availability Statement.

Response4:We have completed the Data Availability Statement in the submission system as follows:The data underlying this study contain sensitive patient information and cannot be made publicly available due to ethical restrictions imposed by the Ethics Committee of Wuxi Second People's Hospital (Approval No. 2024-Y341). Requests for data access should be directed to the Ethics Committee of Wuxi Second People's Hospital (Email: wxeyzzjd@163.com; Tel: +86 510 66618534).

Comment 5(Journal Requirement #6):Please update your submission to use the PLOS LaTeX template.

Response5:We have reformatted the manuscript using the PLOS LaTeX template. The revised manuscript has been compiled accordingly and is now being submitted in compliance with this requirement.

Comment 6(Journal Requirement #7):Please amend your authorship list in your manuscript file to include author Ping Yu, Hong Bian, Jingjing Yan, Zhiyin Zhou, Zhengyu Yang.

Response6:We have added the complete authorship list with the correct order and designated corresponding author to the title page of the revised manuscript, as requested.

Comment 7(Journal Requirement #8) :Please upload a copy of Figure 1, to which you refer in your text on page 5. If the figure is no longer to be included, please remove all reference to it within the text.

Response7:We have removed Figure 1 from the submission. All references to Figure 1 have been deleted from the text (Section 2.3). The search strategy is now fully described in the text, which is sufficient for readers to reproduce the search.

Comment 8 (Journal Requirement #9):If the reviewer comments include a recommendation to cite specific previously published works, please review and evaluate these publications.

Response8:The reviewer did not recommend any specific previously published works for citation. Therefore, no changes were made to the reference list on this point.

Comment 9 (Journal Requirement #10):Please review your reference list to ensure that it is complete and correct. If you have cited papers that have been retracted, please include the rationale for doing so.

Response9:The entire reference list has been rechecked, updated, and standardized to fully meet PLOS ONE Vancouver style requirements. No retracted articles are included in the references.

Response to Reviewer

Reviewer Comment 1:Suggest change title into: "Implementing evidence-based practices for urinary leakage prevention in ICU patients: A JBI-guided baseline review."

Response:We thank the reviewer for this constructive suggestion. We have revised the title accordingly. The new title is:Implementing evidence-based practices for urinary leakage prevention in ICU patients with indwelling catheters: A JBI-guided baseline review.

This change has been made on the title page of the revised manuscript.

Reviewer Comment 2:The study's objective is unclear; please rephrase it.

Response:We apologize for the lack of clarity. We have rephrased the Objective section in the Abstract to state the aim more directly. The revised Objective now reads:

Objective:To develop evidence-based audit indicators for urinary leakage prevention and management in ICU patients with indwelling catheters, to evaluate current clinical compliance with these indicators, and to analyze barriers and facilitators for evidence implementation using the JBI Clinical Evidence Application Model as a theoretical framework.

Reviewer Comment 3:The results in numbers must be linked to clinical or organizational meaning.

Response: We agree with the reviewer. We have added brief interpretive statements in the Results section (3.2) to clarify the clinical relevance of the quantitative findings. The following text has been added:

"The low compliance rates across multiple indicators reveal substantial gaps between current clinical practice and best evidence. These gaps increase the risk of urethral injury, catheter-related infection, leakage, pressure injury, and prolonged hospital stay. Improving adherence to evidence-based protocols is critical to enhancing patient safety and care quality in the ICU."

The relevant in-depth analyses are further elaborated in the Discussion section (4.2).

Reviewer Comment 4:Reordering the sources to conform to the PLOS guidelines.

Response:We have carefully checked, rearranged, and reformatted all references to fully comply with PLOS ONE’s Vancouver reference style. Author format, title case, punctuation, DOI, and citation order have been uniformly revised.

We sincerely hope that the revised manuscript now meets the publication standards of PLOS ONE. Thank you again for your professional and valuable guidance.

Sincerely,

The Authors

The Second People's Hospital of Wuxi, Jiangsu, China

Attachment Submitted filename: 1.Response to Reviewers.docx

# Editor report: Decision Letter 1

**DOI:** 10.1371/journal.pone.0351406.r003

**Authors:** Hussam Awayid, Hussam Awayid

27 May 2026

Implementing evidence-based practices for urinary leakage prevention in ICU patients with indwelling catheters: A JBI-guided baseline review

PONE-D-25-60399R1

Dear Dr. Yu,

We’re pleased to inform you that your manuscript has been judged scientifically suitable for publication and will be formally accepted for publication once it meets all outstanding technical requirements.

Within one week, you’ll receive an e-mail detailing the required amendments. When these have been addressed, you’ll receive a formal acceptance letter and your manuscript will be scheduled for publication.

An invoice will be generated when your article is formally accepted. Please note, if your institution has a publishing partnership with PLOS and your article meets the relevant criteria, all or part of your publication costs will be covered. Please make sure your user information is up-to-date by logging into Editorial Manager at Editorial Manager® and clicking the ‘Update My Information' link at the top of the page. For questions related to billing, please contact billing support.

If your institution or institutions have a press office, please notify them about your upcoming paper to help maximize its impact. If they’ll be preparing press materials, please inform our press team as soon as possible -- no later than 48 hours after receiving the formal acceptance. Your manuscript will remain under strict press embargo until 2 pm Eastern Time on the date of publication. For more information, please contact onepress@plos.org.

Kind regards,

Hussam sami Awayid, Ph.D.

Academic Editor

PLOS One

Additional Editor Comments (optional):

Reviewers' comments:

# Editor report: Acceptance letter

**DOI:** 10.1371/journal.pone.0351406.r004

**Authors:** Hussam Awayid, Hussam Awayid

PONE-D-25-60399R1

PLOS One

Dear Dr. Yu,

I'm pleased to inform you that your manuscript has been deemed suitable for publication in PLOS One. Congratulations! Your manuscript is now being handed over to our production team.

At this stage, our production department will prepare your paper for publication. This includes ensuring the following:

* All references, tables, and figures are properly cited

* All relevant supporting information is included in the manuscript submission,

* There are no issues that prevent the paper from being properly typeset

You will receive further instructions from the production team, including instructions on how to review your proof when it is ready. Please keep in mind that we are working through a large volume of accepted articles, so please give us a few days to review your paper and let you know the next and final steps.

Lastly, if your institution or institutions have a press office, please let them know about your upcoming paper now to help maximize its impact. If they'll be preparing press materials, please inform our press team within the next 48 hours. Your manuscript will remain under strict press embargo until 2 pm Eastern Time on the date of publication. For more information, please contact onepress@plos.org.

You will receive an invoice from PLOS for your publication fee after your manuscript has reached the completed accept phase. If you receive an email requesting payment before acceptance or for any other service, this may be a phishing scheme. Learn how to identify phishing emails and protect your accounts at https://explore.plos.org/phishing.

If we can help with anything else, please email us at customercare@plos.org.

Thank you for submitting your work to PLOS ONE and supporting open access.

Kind regards,

PLOS ONE Editorial Office Staff

on behalf of

Dr. Hussam sami Awayid

Academic Editor

PLOS One
